# Ultrasound-guided acupuncture therapy in Korea: advancing traditional practices with new technology

**DOI:** 10.3389/fmed.2025.1544618

**Published:** 2025-03-26

**Authors:** Sang-ho Lee, You Suk Youn, Min Chul Kim, Junghum Sun, Donghyon Ha, Tae-Hun Kim

**Affiliations:** ^1^Chamjalham Hospital of Korean Medicine, Seoul, Republic of Korea; ^2^Chamjalham Hospital of Korean Medicine, Suwon, Republic of Korea; ^3^Korean Medicine Clinical Trial Center, Korean Medicine Hospital, Kyung Hee University, Seoul, Republic of Korea

**Keywords:** ultrasound-guided acupuncture, Korean Medicine, acupuncture, Korean traditional medicine, ultrasonography, interventional ultrasonography

## Abstract

Ultrasound-guided acupuncture is a novel technique that utilizes ultrasonography to visualize needle placement in real-time, enhancing precision and reducing adverse events. In South Korea, the dual medical system has led to disputes over Korean Medicine doctors’ use of biomedical devices. However, in 2022, the Supreme Court ruled that Korean Medicine doctors’ use of ultrasound devices is not illegal, enabling broader clinical application. Ultrasound-guided acupuncture involves inserting a needle to a precise depth to reach targeted tissue while applying manual techniques based on ultrasound-guided injection therapy principles. This technique requires ultrasonography with a linear probe and is commonly used to treat musculoskeletal conditions, such as shoulder pain. By improving accuracy, ultrasound-guided acupuncture enhances the effectiveness and safety of both traditional acupuncture and emerging techniques like acupotomy and pharmacopuncture. However, its clinical use remains limited due to the lack of national health insurance coverage and insufficient clinical evidence. To establish its efficacy and safety, comparative clinical trials should first focus on musculoskeletal disorders, pain management, and neurological conditions, which are among its most frequent applications in Korea.

## 1 Introduction

Acupuncture is a therapeutic technique with thousands of years of history marked by continuous transformations and advancements in its principles, methods, and therapeutic tools. The theory of acupuncture also has been developed for increasing efficacy and ensuring the safety of the practice (1). In particular, acupuncture theory is being reinterpreted from a biomedicine perspective with the growing use of acupuncture in Europe, including the UK and United States (2). The scope of acupuncture treatment has expanded with the development and application of new techniques such as pharmacopuncture (3), bee venom therapy (4), acupotomy (5), and thread-embedding therapy (6), and these new acupuncture methods represent a significant trend in promoting human health and well-being.

In Korea, the medical system operates as a dual structure in which Korean and Western medicine coexist institutionally. One major issue is the restrictions imposed on Korean Medicine doctors using new biomedical devices (7). Korean Medicine doctors have used it to diagnose and treat diseases. However, the claim that the use of ultrasound was beyond the professional scope of Korean Medicine doctors led to incidents of prosecution of Korean Medicine doctors from 2010 to 2012 over the use of ultrasound diagnostic devices in their clinics. A series of court battles, from the initial trial in 2016 to the Supreme Court ruling in December 2022, finally decided that usage of ultrasound diagnostic devices by Korean Medicine doctors did not violate medical laws (8). This rule allowed Korean Medicine doctors to use ultrasound actively in various aspects of clinical practice. One such application is in ultrasound-guided acupuncture.

Ultrasound-guided injection or needling therapy is commonly used in biomedicine, which aids practitioners in visual confirmation of legion of the disease and accurate injection of medications to maximize treatment efficacy and safety. Various medical fields, including orthopedics, utilize this technique (9) which inspired Korean Medicine doctors to utilize ultrasound in acupuncture to maximize the effectiveness and safety of the treatment.

Needling therapy using ultrasound has primarily been applied within the domain of biomedicine. Specifically, it has been more frequently utilized for deep needling aimed at stimulating trigger points rather than in the context of classic acupuncture. Moreover, research on ultrasound-guided acupuncture has been conducted predominantly in China rather than Korea, with interventions typically involving manual acupuncture, electroacupuncture, or warm needling (10). In contrast, reports from Korea indicate that ultrasound-guided acupuncture is mainly employed in pharmacopuncture or acupotomy and is often used to address not just musculoskeletal diseases but also joint or peripheral nerve disorders. Notably, studies have reported that ultrasound-guided acupuncture can reduce the number of needling sessions and enhance safety (11). This has led to a growing interest in Korea not only in the efficacy but also in its potential to improve the safety of acupuncture treatments, with ultrasound-guided techniques seen as a valuable tool.

According to a recently published biometric analysis of research on ultrasound-guided acupuncture, the number of clinical studies is reported to be increasing (12). However, most of these studies have been conducted outside of Korea and primarily focus on clinical trials evaluating its efficacy. In contrast, research conducted in Korea on ultrasound-guided acupuncture has largely focused on assessing the safety and efficacy of the intervention for specific conditions, without providing a comprehensive overview of its detailed procedures or clinical applications in practice (13). As a result, there are limitations in accurately reflecting the current state of ultrasound-guided acupuncture in Korea. Additionally, studies that present expert opinions or surveys of Korean Medicine doctors offer insights into the clinical applications of this treatment, its advantages, and the factors necessary for increasing its future adoption (14, 15). However, detailed information on how the procedure is performed, the extent of its use across different medical institutions, and its real-world clinical implementation remains insufficient. Consequently, there is a lack of practical guidance for clinicians interested in adopting this treatment, making it difficult for them to rely on existing studies when integrating it into their practice. Addressing these gaps is crucial as a foundation for future high-quality interventional studies aimed at establishing robust evidence for ultrasound-guided acupuncture.

In this brief perspective article, we present an overview of the principal concept of ultrasound-guided acupuncture, examine its current application status, outline treatment methods, and discuss research and clinical practice directions in the future. This commentary aims to provide a snapshot of the clinical status of ultrasound-guided acupuncture currently practiced in Korea. It includes a descriptive analysis of its use and an introduction to the procedures through treatment cases at a Korean Medicine hospital in Seoul. The data were analyzed from hospital records between January 1, 2023, and May 31, 2024.

## 2 Principle concept of ultrasound-guided acupuncture and its brief history of usage in Korea

Ultrasound-guided acupuncture can be defined as the application of the main principles of ultrasound-guided injection therapy to acupuncture, which involves live imaging of the position of the needle under the skin while injecting medication, saline, or aspirating joint effusion and applying these techniques to acupuncture (16). Classic acupuncture is not achieved simply by inserting the acupuncture needle at the exact acupuncture points on the skin; it is completed by inserting the needle to a depth that reaches the targeted tissue and performing manual techniques (17). Recently, new types of acupuncture techniques, such as pharmacopuncture, in which distilled herbal ingredients are injected, and acupotomy, using specialized acupuncture needles, have been utilized in clinical practice. However, locating acupuncture points on the body surface is not sufficient to identify the subcutaneous tissues or spaces that are the treatment targets to maximize therapeutic effects and ensure proper placement of acupuncture needles. Careful and skilled needle insertion is required in particular areas where acupuncture has a potentially serious risk of adverse events, such as acupuncture points in the thoracic region (17). Ultrasound-guided acupuncture helps avoid complications in these cases by confirming the subcutaneous spaces or tissues. Following the 2022 ruling of the Supreme Court that using ultrasound does not violate medical law, Korean Medicine doctors received increased education regarding ultrasound and use of ultrasound in clinics (13).

## 3 Utilization of ultrasound-guided acupuncture in Korea: a snapshot in a local Korean Medicine hospital

Our Korean Medicine hospital in Seoul has provided medical services since 2014 and currently serves approximately 40,000 patients annually. Nine Korean Medicine doctors are working at present, and since the introduction of the first ultrasound machine in 2023, ultrasound-guided acupuncture has been performed in four consultation rooms. An analysis of 2,182 new patients (with a total of 13,269 outpatient visits) who visited between January 1, 2023, and May 31, 2024, showed that, excluding those with temporomandibular joint disorders, thoracic spine diseases, internal diseases, facial palsy, and other miscellaneous conditions, approximately 20% of patients (of the total 10,942 outpatient visits) received ultrasound-guided acupuncture. Diseases (or Indications) for ultrasound-guided acupuncture in our hospital include lumbar and cervical disorders, chronic pelvic pain, shoulder disorders, knee pain, ankle disease, and plantar fasciitis. The national health insurance does not cover the cost of ultrasound diagnostic and therapeutic applications, and acupuncture treatment (including pharmacopuncture) is charged 40,000 KRW per session.

## 4 Tools and general procedures for ultrasound-guided acupuncture

An ultrasonography with a linear probe is required for soft-tissue scanning of the musculoskeletal system in ultrasound-guided acupuncture. The models operated in our hospital include the Versana Balance VA (GE), Vscan Air CL (GE), and Sonoimage HS1 (Konica). A linear probe is typically used in ultrasound transducers, operating at frequencies between 6 and 12 MHz to explore subcutaneous or musculoskeletal tissues (13). Instead of classic acupuncture using filiform needles, pharmacopuncture is primarily selected for this treatment, utilizing disposable 26G (5 cm) and 30G (4 cm) needles with an injection of 3–10 cc of saline or pharmacopuncture solution. Other necessary tools include povidone or alcohol swabs for disinfection, bandages to prevent post-needling skin bleeding, ultrasound gels, medical gloves, and dressing sets.

The general procedure for performing ultrasound-guided acupuncture is as follows:

First, the target area for treatment is examined using ultrasonography.

Next, the target area is identified, and the insertion site is disinfected.

The needle is then positioned at the target site, and saline or pharmacopuncture solution is injected.

Finally, the needle is removed (Supplementary Video S1).

## 5 Ultrasound-guided acupuncture for shoulder pain as an example case

**Figure 1:**
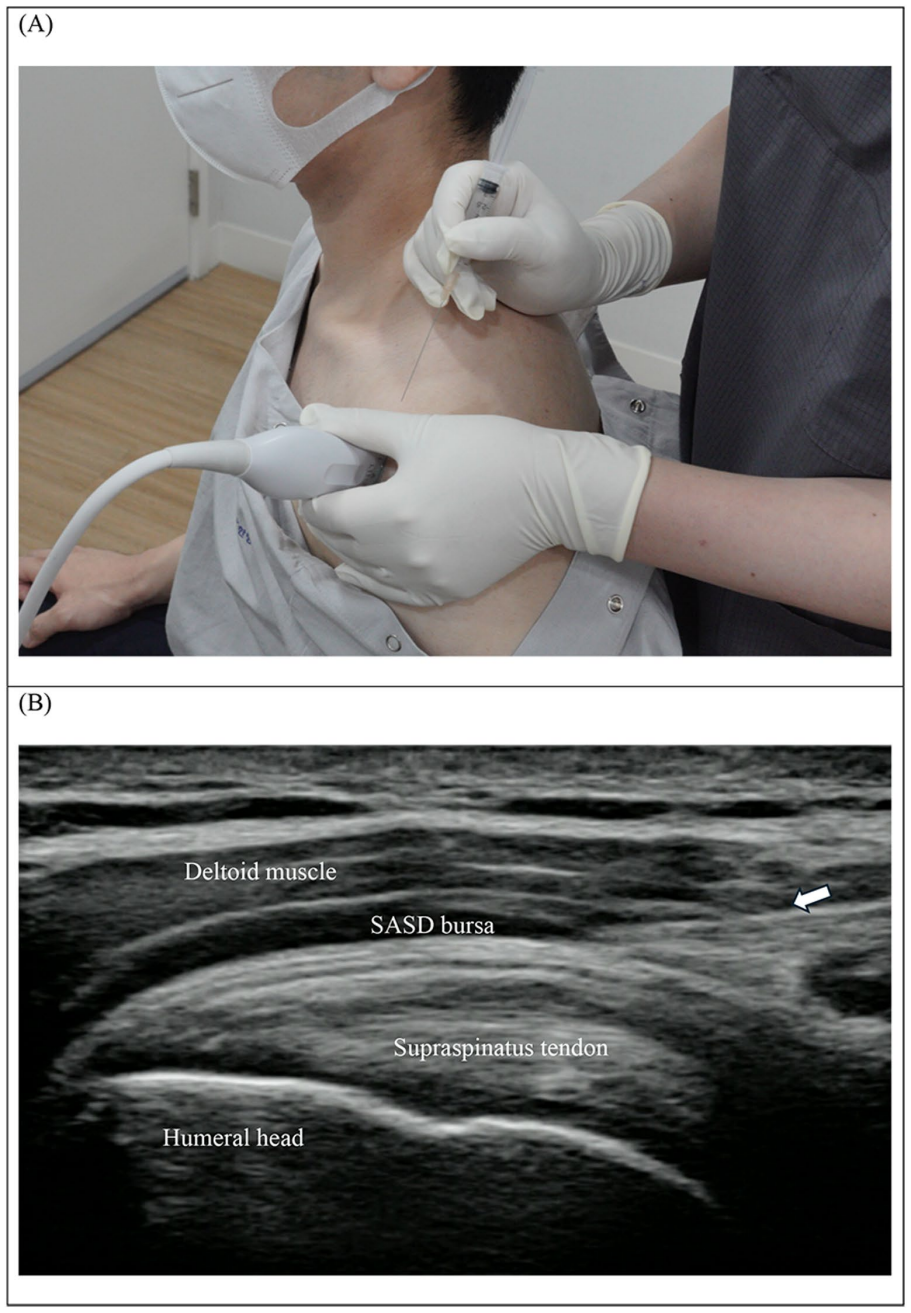
Ultrasound-guided acupuncture for shoulder pain. Pharmacopuncture treatment for shoulder pain. **(A)** The needle located at LI15 point is inserted from the back of the patient. **(B)** The needle (arrow) is placed in the SASD bursa. For this ultrasound-guided acupuncture, Sonoimage HS1 (Konica) was used with a linear probe. It depicts a simulated treatment conducted on a mock patient for demonstrational purposes and does not involve actual patient care. No adverse events were observed after the treatment. SASD, subacromial-subdeltoid.

The shoulder joint has a high degree of mobility, making it highly unstable, leading to shoulder pain due to various reasons, such as rotator cuff tears or injuries, frozen shoulder, and bursitis. Consequently, many patients seek acupuncture treatment, and substantial reported clinical evidence supports the use of acupuncture for shoulder pain (18). The clinical guidelines for acupuncture treatment of shoulder pain in adults recommend using proximal acupuncture points such as LI15, TE14, SI9, GB21, SI14, TE15, and Ashi points, and distal acupuncture points such as LI11, TE5, LI4, SI3, ST38, BL57, and GB34 (18). However, these guidelines only suggest acupuncture points and do not specifically address detailed treatment methods and related information, including the depth and precise targeting points for acupuncture treatments. LI15 is the most used proximal acupuncture point for shoulder pain treatment. In ultrasound-guided acupuncture treatment, while LI15 is located according to manual acupuncture methods using the surface anatomy of the body, the actual treatment target during acupuncture is to position the needle tip within or in the space between the tendon and the subacromial-subdeltoid (SASD) bursa. The position of the needle tip is verified using ultrasonography. For this procedure, the patient is positioned in a modified Crass position, facing the practitioner with the hand of the affected shoulder placed on the hip of the affected side. The supraspinatus tendon is identified by connecting the acromion and greater tubercle of the humerus, and the probe is placed longitudinally along the tendon path. The acupuncture needle (or injection needle for pharmacopuncture) is inserted from superior to inferior (cranial to caudal) and advanced accordingly (Figure 1). Precautions during ultrasound-guided acupuncture include monitoring for strong sensations when the needle stimulates the tendon and checking for excessive pain during the treatments. In addition, sterilization and patient hygiene must be carefully monitored to prevent infection at the insertion site.

## 6 Future of ultrasound-guided acupuncture

As of 2024, ultrasound-guided acupuncture is primarily utilized in pharmacopuncture to treat musculoskeletal disorders in Korea (13). Recent studies have indicated that ultrasound-guided acupuncture is extensively used in China to enhance the effectiveness and safety of acupotomy treatments for conditions such as shoulder pain, knee pain, peroneal nerve palsy, and metatarsal tunnel syndrome (19). It is anticipated that ultrasound-guided acupuncture will significantly enhance safety when targeting acupuncture points around the thoracic region or areas at risk of nerve or blood vessel damage. Additionally, ultrasound-guided acupuncture can expand the scope of techniques used by acupuncture practitioners, such as the aspiration of Baker’s cysts and the removal of joint effusions (20). While ultrasound is harmless to the human body and helps to accurately and safely visualize the structures where the needle is inserted, its current application is limited because the national health insurance does not yet cover ultrasound-guided acupuncture in Korea, thus requiring patients to pay the entire cost. In addition, adequate training is necessary for effectively using ultrasound in acupuncture treatment. As a strategy for establishment of clinical evidence, it is necessary to first conduct comparative clinical trials on musculoskeletal conditions, pain, and neurological disorders, which are frequently treated with ultrasound guided acupuncture in Korea. Gradually, studies evaluating the safety of procedures in areas such as the abdomen, chest, and head and neck should also be carried out. Additionally, cost-effectiveness analyses and the development of clinical guidelines for practitioners should be conducted to facilitate inclusion in the national insurance coverage. Clinical trials comparing the effectiveness with classic acupuncture and conventional therapies will also be required. Additionally, studies are needed to establish clinical evidence by evaluating the safety and effectiveness of ultrasound-guided acupuncture using various interventions such as piliform needles, pharmacopuncture, and acupotomy. Furthermore, there is a need to develop needles specifically designed for ultrasound-guided acupuncture and ultrasound devices tailored to acupuncture applications.

## 7 Clinical implications for Korean Medicine doctors

Currently, there are no official guidelines available for ultrasound-guided acupuncture for Korean Medicine doctors. Therefore, based on our experience, we recommend the following considerations for beginners when applying this technique in clinical practice:

1. Prioritize applying ultrasound-guided acupuncture to conditions such as subacromial bursitis or shoulder pain, as these are suitable starting points.
2. Take precautions to prevent infection before, during, and after the procedure.
3. Since the use of ultrasound-guided acupuncture requires knowledge of ultrasonography operation as well as the anatomical understanding of the targeted area, ensure completion of appropriate training and practical exercises.

## Data Availability

The raw data supporting the conclusions of this article will be made available by the authors, without undue reservation.

## Supplementary material

SUPPLEMENTARY VIDEO S1 Video clip for the detailed procedure of ultrasound-guided acupuncture.
